# Slow conduction in mixed cultured strands of primary ventricular cells and stem cell-derived cardiomyocytes

**DOI:** 10.3389/fcell.2015.00058

**Published:** 2015-09-24

**Authors:** Jan P. Kucera, Yann Prudat, Irene C. Marcu, Michela Azzarito, Nina D. Ullrich

**Affiliations:** ^1^Department of Physiology, University of BernBern, Switzerland; ^2^Department of Physiology and Pathophysiology, Heidelberg UniversityHeidelberg, Germany

**Keywords:** stem cells, ventricular myocytes, cardiac conduction, action potential, conduction velocity

## Abstract

Modern concepts for the treatment of myocardial diseases focus on novel cell therapeutic strategies involving stem cell-derived cardiomyocytes (SCMs). However, functional integration of SCMs requires similar electrophysiological properties as primary cardiomyocytes (PCMs) and the ability to establish intercellular connections with host myocytes in order to contribute to the electrical and mechanical activity of the heart. The aim of this project was to investigate the properties of cardiac conduction in a co-culture approach using SCMs and PCMs in cultured cell strands. Murine embryonic SCMs were pooled with fetal ventricular cells and seeded in predefined proportions on microelectrode arrays to form patterned strands of mixed cells. Conduction velocity (CV) was measured during steady state pacing. SCM excitability was estimated from action potentials measured in single cells using the patch clamp technique. Experiments were complemented with computer simulations of conduction using a detailed model of cellular architecture in mixed cell strands. CV was significantly lower in strands composed purely of SCMs (5.5 ± 1.5 cm/s, *n* = 11) as compared to PCMs (34.9 ± 2.9 cm/s, *n* = 21) at similar refractoriness (100% SCMs: 122 ± 25 ms, *n* = 9; 100% PCMs: 139 ± 67 ms, *n* = 14). In mixed strands combining both cell types, CV was higher than in pure SCMs strands, but always lower than in 100% PCM strands. Computer simulations demonstrated that both intercellular coupling and electrical excitability limit CV. These data provide evidence that in cultures of murine ventricular cardiomyocytes, SCMs cannot restore CV to control levels resulting in slow conduction, which may lead to reentry circuits and arrhythmias.

## Introduction

Stem cell-based therapy represents the new hope for cardiac repair of the injured heart after damage or disease. Recently, major advances have been achieved in this field and provide strong evidence for the high potential of grafted cells to survive and functionally replace diseased cardiomyocytes (Shiba et al., 2012; Chong et al., 2014). For functional integration into host myocardium, stem cell-derived cardiomyocytes (SCMs) must possess similar electrophysiological properties and the ability to establish intercellular connections with host cardiomyocytes in order to contribute to the electrical signal propagation and mechanical function (McCain et al., 2012). Insufficient excitability or defective intercellular coupling may significantly alter electrical conduction and contribute to the development of arrhythmias (Yao et al., 2003; Danik et al., 2004; Liao et al., 2010).

Impulse propagation across the myocardium is determined by various parameters that control myocyte excitability, action potential (AP) properties and intercellular resistance (Kléber and Rudy, 2004; Veeraraghavan et al., 2014). The electrical excitability of a cardiomyocyte depends on the sarcolemmal ion channel repertoire and functional expression of fast voltage-dependent Na^+^ channels, which define the upstroke of the AP (Weidmann, 1955; Berecki et al., 2010). The activity of Na^+^ channels is not only controlled by their expression but is also limited by the resting membrane potential of the myocyte, requiring strongly negative resting membrane potentials for full channel availability. Reduced Na^+^ current (*I*_Na_) may therefore significantly alter the AP upstroke and reduce myocyte excitability.

In order to form a functional syncytium, adjacent myocytes are connected via gap junctions, low resistance channels that enable electrical and metabolic coupling between cells. In ventricular myocytes, gap junctions are predominantly built from connexin 43 (Cx43) subunits. The importance of ventricular gap junctional coupling on AP propagation has been previously established by several studies using Cx43 knockout mouse models (Morley et al., 1999; Eloff et al., 2001; Gutstein et al., 2001a; Vaidya et al., 2001; Beauchamp et al., 2004; van Rijen et al., 2004). These studies showed that decreased Cx43 content reduces conduction velocity (CV). In further studies, the impact of a heterogeneous Cx43 expression on impulse propagation has been demonstrated using *in situ* and *in vitro* models (Gutstein et al., 2001b; Beauchamp et al., 2012). In addition to significantly reducing CV, heterogeneously decreased Cx43 content led to inhomogeneous propagation of wave fronts and conduction blocks, which may be translated into a high pro-arrhythmogenic potential of cells expressing different levels of Cx43. In this context, we have recently shown that SCMs exhibit significantly lower levels of Cx43 both at mRNA and protein levels compared with native primary myocytes, resulting in strongly reduced intercellular coupling of cell pairs (Marcu et al., 2015). Thus, despite the cardiogenic features of SCMs, limitations in the functional formation of electrical intercellular connections may strongly impair their integrative potential in host myocardium and the rescue of electrical activity in the diseased heart.

In the present study, our goal was to investigate AP propagation in heterocellular cardiac tissue simulating cell grafts. To test the hypothesis that CV is reduced in tissue consisting of SCMs or a mixture of SCMs and primary cardiomyocytes (PCMs), we produced cell strands of defined proportions of murine SCMs and PCMs. The electrical properties of cell strands relative to the SCM content were evaluated using microelectrode arrays and SCM excitability was determined by the single cell current clamp technique. In ventricular tissue, the expression pattern and location of gap junctions are organized and concentrated in intercalated discs, thereby defining the principal direction of signal propagation along the longitudinal axis of the myocytes. In cultured PCMs and SCMs, this tissue architecture and axial polarization are absent and therefore, in our experiments, the direction of propagation was forced along cultured cell strands. Experiments were paralleled with simulations using a tissue model that integrates the heterogeneity of the cells, electrical excitability and intercellular coupling.

Confirming our hypothesis, here we provide evidence that co-culture of SCMs with PCMs results in reduced CVs, potentially limiting the safe application of SCMs in the clinical setting.

## Methods

### Co-cultured strands on microelectrode arrays

GFP-tagged murine embryonic SCMs were commercially obtained from Axiogenesis (Cologne, Germany). The process of differentiation from pluripotent SC to the cardiac phenotype took 15 days, then cells were frozen, stored and shipped in liquid nitrogen. After thawing SCMs were first cultured in puromycin-containing proprietary medium (Axiogenesis) to inhibit growth of non-cardiac stem cells and to select the GFP-expressing cells. The pac gene expression for puromycin resistance was under control of the mouse α-MHC promoter (Axiogenesis). For co-cultures, cells were detached after 48 h using trypsin and combined with freshly dissociated PCMs.

The PCMs were dissociated as described previously (Beauchamp et al., 2004) from the hearts of wild-type murine fetuses at postcoital day 19. The animals were handled according to the ethical principles and guidelines of the Swiss Academy of Medical Sciences. The protocols were independently reviewed and approved by the Commission of Animal Experimentation of the Cantonal Veterinary Office of the Canton of Bern, Switzerland. The suspensions of dissociated PCMs were plated for 2 h to minimize myofibroblast content. After cell counting using a Neubauer chamber, the suspensions of PCMs and SCMs were mixed in predefined proportions of 0 (only PCMs), 1/3, 2/3, and 1 (only SCMs). Cells were seeded on microelectrode arrays (Centre Suisse d'Électronique et de Microtechnique, Neuchâtel, Switzerland) at a total seeding density of 3.5·10^5^/cm^2^ to form 150 to 200 μm wide patterned strands using photolithographic techniques, as detailed previously (Kondratyev et al., 2007). The microelectrode arrays were mounted in custom-made culture chambers. Each strand (length: 0.7 cm) was grown over a row of 12 extracellular electrodes (interelectrode spacing: 0.5 mm; diameter 40 μm) and two stimulation dipoles.

The strands were incubated in M199 medium (with Hanks' salts; Sigma-Aldrich, Buchs, Switzerland) supplemented with streptomycin (20 mg/L, Oxoid, Pratteln, Switzerland), penicillin (20000 U/L, Oxoid, Pratteln, Switzerland) and bromodeoxyuridine (100 μmol/L, Sigma-Aldrich, Buchs, Switzerland). The latter was used to inhibit proliferation of myofibroblasts (Beauchamp et al., 2004).

### Extracellular recording of impulse propagation

Electrophysiological recordings were conducted 2 days after seeding. The M199 medium was replaced with Hanks' balanced salts solution (Sigma-Aldrich, Buchs, Switzerland), the microelectrode arrays were connected to a custom-made amplifier array (gain: × 1000) and the system was placed in an incubator (36°C, 0.9% CO_2_). Experiments were started after a stabilization period of ≥60 min.

The strands were paced at a cycle length (CL) of 300 ms with biphasic voltage pulses at 1.5–2 times threshold during a period of 1 min. Conduction characteristics (velocity, heterogeneity) were assessed during steady state at the end of this period. Activation times were identified at each electrode by the occurrences of the minima of the first time derivatives of the extracellular electrograms. CV was computed for every AP by linear regression of activation time vs. distance. After having verified that stimuli were captured and that no spontaneous activity was present, CV was then determined for every strand as the mean CV during the last 10 s of the 1-min pacing period at CL = 300 ms. Conduction heterogeneity was estimated by computing CVarCT_0.5 mm_, the coefficient of variation (ratio of standard deviation to the mean) of conduction times between successive electrodes (spaced 0.5 mm from each other). This marker is 0 in the case of perfectly uniform conduction and increases with the degree of heterogeneity.

To estimate the refractory period, CL was progressively decreased on a beat-to-beat basis from 300 to 50 ms at a rate of 50 ms/min. The effective refractory period was quantified as the interpulse interval between the last two propagated APs immediately before the first stimulation failure or conduction block.

### Quantification of the proportion of SCM in the preparations

Fluorescence microphotographs (2048 × 1536 pixels, spatial resolution 0.34 μm, 8 bit) of the preparations were taken to ascertain the proportion of GFP-tagged SCMs in the cultured strands and the variability of this proportion. Images were first converted to grayscale and pixel intensity was normalized to a range between 0 (lowest intensity) and 1 (highest intensity). The images were then filtered using a median filter on 3 × 3 neighborhoods followed by Gaussian low-pass filtering using a 3 × 3 kernel (standard deviation: 0.5 pixels). Pixel intensity was then adjusted and rescaled such that pixels having a value greater than three times the mean pixel value were set to 1 and pixels with a value less than the median value were set to 0. Pixel values between these extremes were scaled linearly between 0 and 1. In order to discriminate between dark pixels (no fluorescence) and bright pixels (fluorescence), images were segmented using the K-means clustering algorithm (Mignotte, 2008) using two clusters, resulting in a binary classification of the pixels (bright vs. dark). This analysis was conducted on a region of interest (ROI) comprising the cell strands and excluding clusters of presumably dead cells still adhering to the preparation. Individual dark pixels and dark gaps in the resulting binary image were filled based on a four-connected neighborhood. The level of fluorescence was then quantified as the proportion of bright pixels in the ROI. The proportion of SCMs in the preparations was finally quantified by renormalizing this fluorescence level to the mean fluorescence levels of preparations consisting of either PCMs or SCMs only.

### Assessment of SCMs excitability using patch clamp recordings

SCM were seeded at low density on fibronectin-coated glass bottom dishes (MatTek Corp., Texas, US) and used between day 3 and day 7 after seeding. Patch pipettes were pulled from borosilicate glass capillaries using a horizontal DMZ puller (Zeitz Instruments, Martinsried, Germany), resulting in a tip resistance of 3–6 MΩ. APs were acquired from single SCMs at 10 kHz sampling frequency in the current clamp mode of the patch clamp technique controlled by a HEKA EPC10 amplifier and the Patchmaster software (HEKA Elektronik, Lambrecht, Germany). The bath solution contained (in mmol/L): 140 NaCl, 5.4 KCl, 1.8 CaCl_2_, 1.1 MgCl_2_, 5 HEPES, 10 Glucose. The pipette solution contained (in mmol/L): 30 KCl, 90 KAsp, 5 MgCl_2_, 2 CaCl_2_, 5 EGTA, 10 HEPES, 5 Na_2_ATP. The whole cell modus was first established in the voltage clamp mode before switching to current clamp using the bridge function of the amplifier. Cells were stimulated at 0.4 Hz using depolarizing 3–5 ms current pulses just above the threshold intensity to ensure that the stimulation artifact did not interfere with the steepest part of the upstroke. APs were analyzed in Origin software (OriginLab Corp., Northampton MA). Excitability was estimated from AP upstroke velocities (dV/dt_max_), which was determined by differentiating the initial 15–20 ms of the AP including the stimulation artifact.

### Computer simulations of conduction

Simulations of AP propagation were run in randomly generated two dimensional tissues (3000 μm long and 150 μm wide strands) mimicking cardiac cellular architecture. The procedure to construct the simulation of tissues and the numerical methods are detailed in a previous publication (Prudat and Kucera, 2014). Ion currents were formulated according to the Luo–Rudy phase I model (Luo and Rudy, 1991) with adjusted Na^+^ current conductance (gNa_max_), Ca^2+^ current conductance and kinetics, and K^+^ current conductance. The intercellular coupling level was adjusted to produce a longitudinal CV of 34.9 cm/s corresponding to the mean CV in the present control experiments in pure PCM strands. The excitability (gNa_max_) of SCMs as well as the levels of coupling between SCMs and between SCMs and PCMs were adjusted according to experimental data as detailed in the Results Section.

Propagation was followed by identifying the earliest activation time (EAT, determined at dV/dt_max_) along the *x*-direction and CV was computed by linear regression of EAT vs. *x* between 25 and 75% of strand length to exclude stimulation and boundary artifacts. For every proportion of PCMs/SCMs and for every setting of excitability and intercellular coupling, 20 simulations were conducted with different random realizations of the cellular architecture.

### Statistics

All analyses and simulations were performed using MATLAB software (The Math Works, Natick, MA, USA). Data are presented as mean ± standard deviation (SD). Comparison between groups was performed using analysis of variance (ANOVA) after checking for the normality of distributions using the Anderson–Darling test. Differences were considered significant at *p* < 0.05.

To characterize the relationship between CV and the proportion of SCMs (p_SCM_, in the interval [0, 1]), CV was fitted using a least squares algorithm with a linear function (CV = a_1_ · p_SCM_+a_0_) and a quadratic function (CV = a_2_ · p^2^_SCM_+a_1_ · p_SCM_+a_0_). To ascertain whether the dependence of CV on p_SCM_ was better described by a quadratic function than a linear function, we examined whether zero was included in the 95% confidence interval (95% CI) of the quadratic coefficient a_2_. In this case, the relationship between CV and p_SCM_ was considered to be non-linear, with a_2_ reflecting the curvature of the CV vs. p_SCM_ function. Otherwise, we considered that a linear function was sufficient to describe the dependence of CV on p_SCM_.

## Results

### The SCM proportion in cultured strands matches that determined at the time of seeding

Since PCMs and GFP-tagged SCMs may exhibit different viabilities and attach with different strengths on the culture substrate, we first evaluated whether the proportion of PCMs and SCMs corresponded to the proportion that was predefined at the time of seeding.

**Figure 1:**
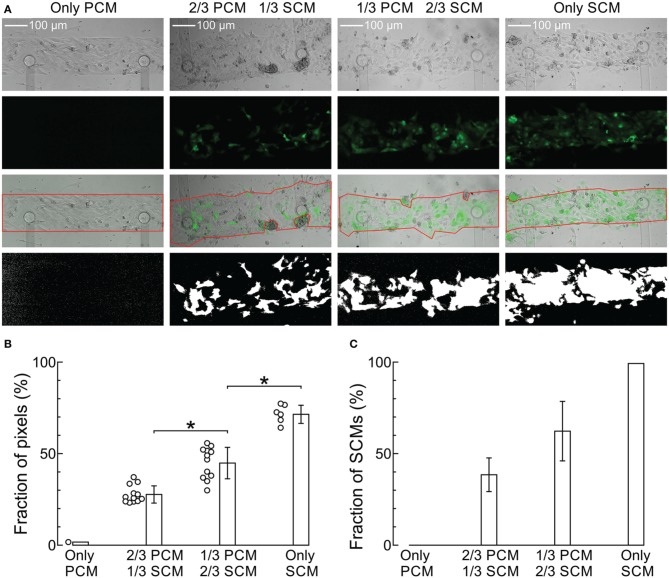
**Imaging of the cultured strands and quantification of the proportion of SCMs. (A)** Phase contrast (top row), fluorescence (second row), overlay with ROI (third row) and binary fluorescence (fourth row) images of strands seeded with increasing predefined densities of GFP-tagged SCMs (labels on top). The respective proportions of white pixels in the binary images were 1.7, 23.7, 51.3, and 77.0%, respectively. **(B)** Analysis of white pixel proportion in all images. ^*^p < 0.05 (ANOVA). **(C)** Proportion of SCMs in the four density groups after renormalization of the data in **(B)** and error propagation.

Figure 1A shows phase contrast and fluorescence microphotographs of co-cultured strands seeded at SCM proportions of 0 (only PCMs), 1/3, 2/3, and 1 (only SCMs) as well as overlay pictures and binary fluorescence images obtained by digital processing. With increasing SCM proportion, a higher fraction of the cells exhibited fluorescence with an increased proportion of white pixels in the binary images (calculated in the region of interest marked in red; analysis shown in Figure 1B). The fluorescence of GFP-tagged SCMs was heterogeneous and very weak in some cells in the 100% SCM preparations. Consequently, the proportion of white pixels in the processed images did not reach 100%. Therefore, the data in Figure 1B were renormalized from 0 (only PCMs) to 100% (only SCMs), as shown in Figure 1C. The error bars in Figure 1C were obtained by propagating the error in the data shown in Figure 1B and represent an estimate of the error on the true proportion of SCMs in the preparations. Figure 1C demonstrates that the proportion of SCMs obtained via image analysis corresponded to the proportion determined at seeding. However, some uncertainty remained regarding the exact proportion of SCMs in the preparations. Regarding cell size, no manifest differences between SCMs and PCMs were observed.

### Conduction becomes slow and heterogeneous with increasing proportions of SCMs

**Figure 2:**
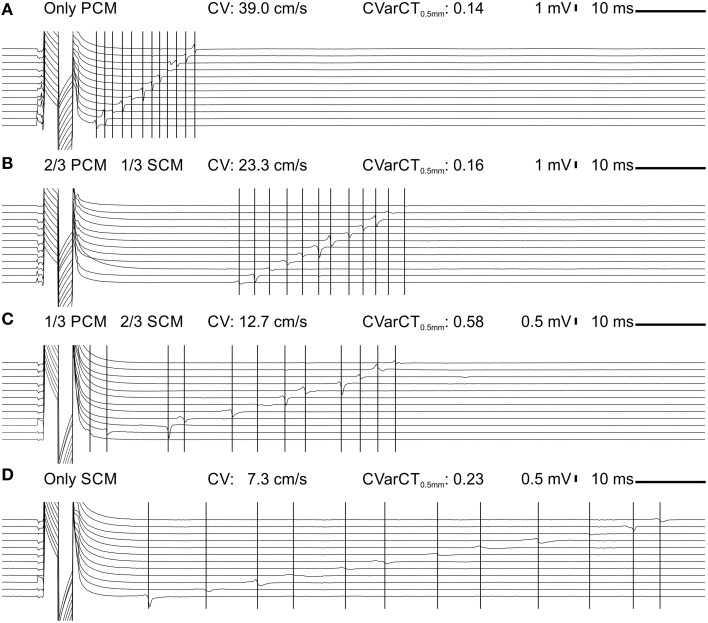
**Extracellular signals during impulse propagation in cultured strands grown over a row of 12 electrodes (0.5 mm spacing) for preparations with an increasing proportion of SCMs (A: 0; B: 1/3; C: 2/3; D:1)**. Note the different voltage scale in **(C,D)**. The large deflection on the left of the traces is the stimulation artifact. The vertical lines denote corresponding activation times during impulse propagation.

Figure 2 shows representative extracellular electrograms recorded from strands grown on a row of 12 electrodes spaced 0.5 mm from each other. The strands were paced at a CL of 300 ms. CV decreased with increasing proportion of SCMs (Figures 2A–D). In addition, conduction appeared heterogeneous in co-cultures of PCMs and SCMs. This heterogeneity was quantified by CVarCT_0.5 mm_ and can be appreciated visually by the irregular intervals between activation times (marked with vertical lines) at successive electrodes (most prominent in Figure 2C for a strand with 1/3 PCMs and 2/3 SCMs).

**Figure 3:**
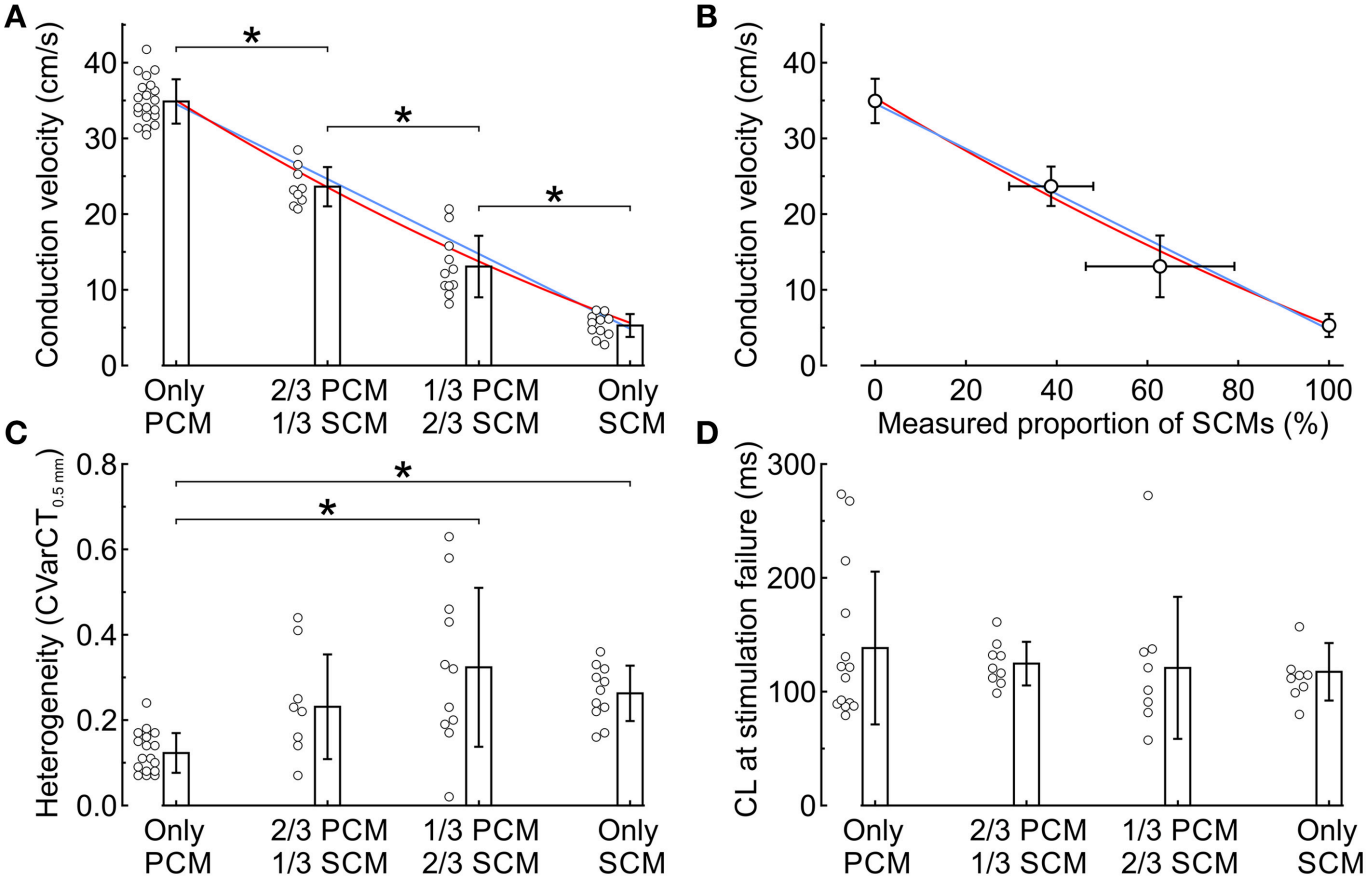
**Electrophysiological characteristics in co-cultured strands of PCMs and SCMs as a function of the relative proportion of the two cell types. (A)** Conduction velocity vs. seeded proportion of SCMs. The blue line and the red curve represent linear and quadratic fits to the data, respectively. **(B)** Conduction velocity vs. measured proportion of SCMs. The blue line and the red curve represent linear and quadratic fits to the data, respectively. **(C)** Heterogeneity index CVarCT_0.5mm_. **(D)** Cycle length at first stimulation failure during the descending ramp pacing protocol. ^*^p < 0.05 (ANOVA).

Data from all experiments and corresponding analyses are presented in Figure 3. CV was significantly different across the four SCM proportion categories and decreased gradually from 34.9 ± 2.9 (only PCMs, *n* = 21) to 5.5 ± 1.5 cm/s (only SCMs, *n* = 11). The dependence of CV on the proportion of SCMs defined at seeding was slightly non-linear (Figure 3A), as reflected by a quadratic coefficient just barely different from 0 at the 95% confidence level (a_2_ = 7.8 cm/s; 95% CI: 0.2-15.5 cm/s). The decrease of CV with the proportion of SCMs is also apparent in Figure 3B, where CV is represented as a function of the proportion of SCMs quantified using image analysis (see Figure 1). However, CV was not better described by a quadratic fit than by a linear fit (a_2_ = 6.3 cm/s; 95% CI: −122.5-135.1 cm/s), possibly because of the large variability of the measured proportion of SCMs in the preparations. Figure 3C shows that conduction was more heterogeneous in co-cultures with 2/3 of SCMs than in control pure PCM preparations. Conduction was also more heterogeneous in pure SCM strands than in pure PCM strands. As shown in Figure 3D, no difference was observed between the four categories in terms of effective refractory period (estimated as the CL at stimulation failure during the protocol in which CL was decreased progressively). Of note, conduction blocks were not observed in these experiments although conduction was more heterogeneous in strands of co-cultured PCMs and SCMs.

### Two populations of SCMs with distinct levels of excitability

**Figure 4:**
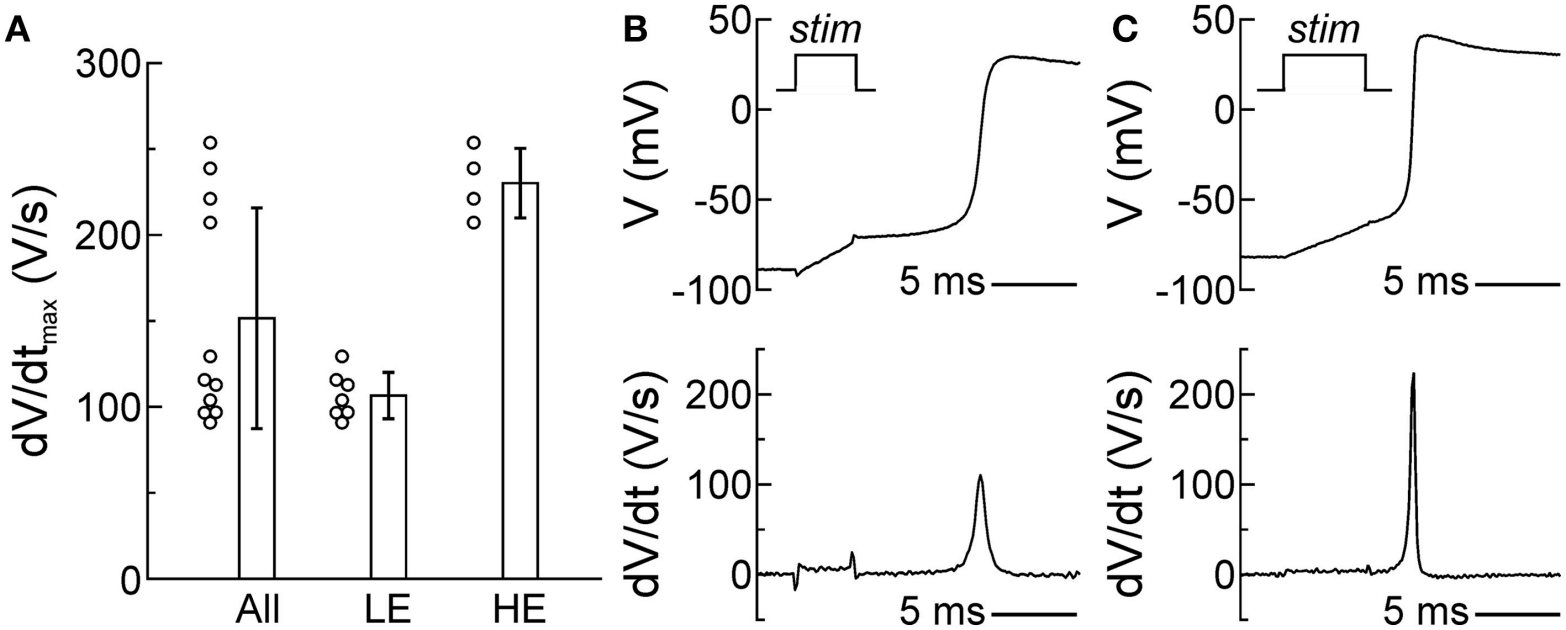
**Two subpopulations of SCMs with distinct levels of excitability. (A)** Distribution of dV/dt_max_ of all cells (left, *n* = 11). The distribution was bimodal and poorly reflected by its mean ± SD, revealing two subpopulations with different levels of excitability (middle: low excitability; right: high excitability). **(B,C)** Example AP upstrokes and dV/dt in a cell with low **(B)** and high excitability **(C)**. The stimulation pulse (*stim*) of the current clamp is indicated in the upper panels and corresponds temporally with the stimulation artifacts in the voltage traces and the calculated derivatives.

Membrane excitability, determined essentially by the density of the voltage-gated Na^+^ current, is a major determinant of CV. Therefore, we evaluated the excitability of SCMs by assessing the dV/dt_max_ of APs recorded using the patch clamp technique in isolated SCMs. In single cells, dV/dt_max_ reflects the peak current during the upstroke. As shown in Figure 4A, dV/dt_max_ was 151 ± 64 V/s in 11 investigated cells; however, the distribution of dV/dt_max_ was bimodal and poorly reflected by its mean and standard deviation. Out of 11 cells, dV/dt_max_ was < 150 V/s in about two thirds of them (7 cells, low excitability (LE): dV/dt_max_: 107 ± 13 V/s; example upstroke in Figure 4B) whereas it was >150 V/s in about one third (4 cells, high excitability (HE): dV/dt_max_: 230 ± 20 V/s; example upstroke in Figure 4C). The SCMs exhibited a resting membrane potential ≤ −75 mV. Resting membrane potential was not statistically different between LE and HE SCMs. Thus, SCMs consisted of two populations with distinct levels of excitability.

### Computer simulations of conduction

To gain a deeper insight into conduction characteristics and the determinants of CV in the co-cultures, we employed a two-dimensional computational framework to generate a model of cardiac tissue strands with stochastic cellular distributions. In this framework, the properties of every individual cell can be specified (Prudat and Kucera, 2014). Because the estimated effective refractory period did not differ between SCM, PCM, and mixed cell strands (Figure 3D) and because SCMs exhibited a resting membrane potential ≤ −75 mV (Figures 4B,C), the same ionic model (Luo and Rudy, 1991) was used for both cell types, except for gNa_max_ which was adjusted as shown in Table 1 to reproduce in single cells the same mean dV/dt_max_ as found in LE-SCMs and HE-SCMs.

**Table 1:** **gNa_max_ of PCMs and the two types of SCMs with LE and HE used in the computer model**.

**Cell type**	**gNa_max_(mS/cm^2^)**
PCM	13.5
LE-SCM	4.5
HE-SCM	11.5

In the simulations, we first observed that a reduction of gNa_max_ from 13.5 mS/cm^2^ (level of PCMs) to 4.5 mS/cm^2^ (level of LE-SCMs) in all cells caused only a moderate reduction of CV from 35.1 ± 0.1 to 22.2 ± 0.1cm/s (*n* = 20 realizations of the cell network). Thus, reducing excitability alone was not sufficient to account for the slow conduction observed experimentally in SCM strands. Consequently, in a second step, the gap junctional coupling level of SCMs was decreased to match the CVs measured in pure SCM cultured strands. To obtain a CV of 5.6 ± 0.1 cm/s corresponding to the CV measured in cultures of 100% SCMs, it was necessary to decrease intercellular coupling by approximately 96% in LE-SCM strands (to 0.0385 relative to PCMs) and by 98% in HE-SCM strands (to 0.0205 relative to PCMs). This finding indicates that the coupling between SCMs is considerably lower than that between PCMs.

Simulations were then run in strands with different proportions of SCMs. The SCMs consisted of LE-SCMs, HE-SCMs, or a mixture of 2/3 LE-SCMs and 1/3 HE-SCMs corresponding roughly to the proportion observed in patch clamped isolated cells. For these simulations, heterocellular coupling was set as the minimum gap junctional coupling level of the corresponding cell types, assuming here that net coupling is determined by the cell expressing the lowest amount of connexins. This assumption is based on the consideration that each cell has to contribute one hemichannel for the formation of one full functional intercellular channel. The heterocellular coupling levels are presented in matrix form in Table 2.

**Table 2:** **Intercellular gap junctional coupling levels in the computer model**.

**Relative coupling**	**PCM**	**LE-SCM**	**HE-SCM**
PCM	1	0.0385	0.0205
LE-SCM	0.0385	0.0385	0.0205
HE-SCM	0.0205	0.0205	0.0205

*Homocellular coupling values are on the diagonal. Values for heterocellular coupling were set by assuming that net coupling is determined by the cell expressing the lowest amount of connexins*.

**Figure 5:**
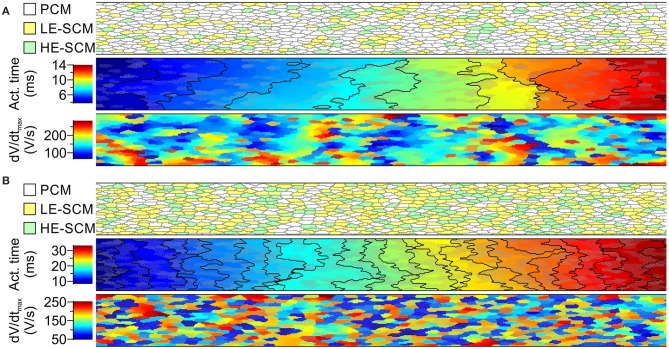
**Simulations of action potential propagation in randomly generated strands combining PCMs and SCMs in proportions of 2/3 PCMs and 1/3 SCMs (A) and 1/3 PCMs and 2/3 SCMs (B)**. Each panel shows a map of cellular architecture (top), the corresponding activation map with isochrones (interval: 1 ms) and the map of dV/dt_max_. The SCMs were distributed randomly with a ratio of 2:1 between SCMs with low and high excitability. SCMs are shown in color (yellow/green) or with an overlaid dotted texture. Overall conduction velocities: 17.7 cm/s in **(A**) 7.0 cm/s in **(B)**.

Figure 5 illustrates AP propagation and dV/dt_*max*_ in a randomly generated strand combining 2/3 PCMs and 1/3 SCMs (Figure 5A) and 1/3 PCMs and 2/3 SCMs (Figure 5B). The SCMs were distributed between LE-SCMs and HE-SCMs in a ratio of 2/3:1/3. The corresponding overall CVs amounted to 17.7 cm/s and to 7.0 cm/s, respectively. Furthermore, the isochronal activation maps and plots show that conduction was heterogeneous in such strands, as reflected by irregular and frequently oblique isochrones. The maps of dV/dt_*max*_ show that combining the different cell types led to an enormous variation of dV/dt_max_, even within a given cell type.

**Figure 6:**
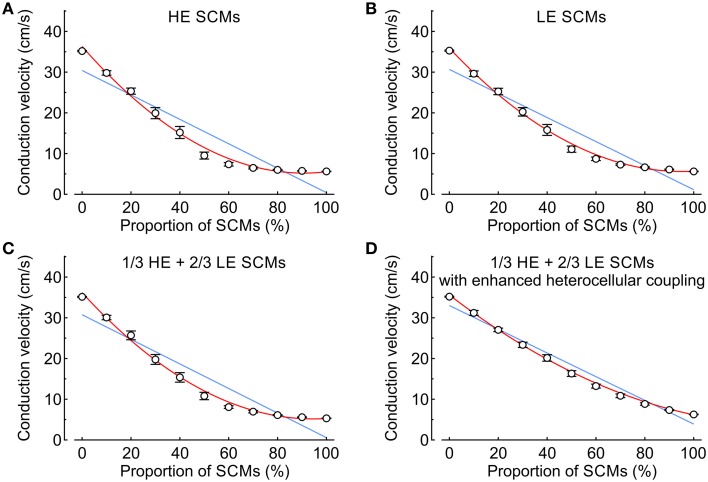
**Conduction velocity vs. proportion of SCMs (0–100% in steps of 10%) in simulated strands (mean ± SD; ***n*** = 20 for every data point)**. Blue lines and red curves represent linear and quadratic fits to the data, respectively (fitting parameters in Table 3). **(A)** Strands combining PCMs and SCMs with high excitability. **(B)** Strands combining PCMs and SCMs with low excitability. **(C)** Strands in which the SCMs were distributed with a ratio of 2:1 between low and high excitability SCMs. **(D)** Same as **(C)**, but with a fourfold increase of the heterocellular coupling conductance between SCMs and PCMs.

In the subsequent analysis presented in Figure 6, CV was examined as a function of the proportion of SCMs using either HE-SCMs (Figure 6A), LE-SCMs (Figure 6B) or HE-SCMs and LE-SCMs in a ratio of 1/3:2/3 (Figure 6C). The relationship between the proportion of SCMs and CV was clearly non-linear in the simulations, in contrast to experimental observations (corresponding fitting parameters with 95% CIs are provided in Table 3). This non-linear behavior of CV motivated us to test the effect of a speculative upregulation of heterocellular gap junctional coupling in the tissue model. Therefore, in an additional series of simulations, coupling between SCMs and PCMs was increased fourfold and coupling between LE-SCMs and HE-SCMs was increased to the level of LE-SCMs, as detailed in Table 4. As shown in Figure 6D, this enhancement of heterocellular coupling decreased the quadratic coefficient to a value more comparable to that in experimental data (see Table 3). Thus, enhancing heterocellular coupling decreased the curvature of the relationship between CV and the proportion of SCMs in a manner agreeing with the experimental data. Finally, we noted that with either coupling paradigm, conduction block never occurred in the simulations.

**Table 3:** **Fitting parameters for CV (in cm/s) for the simulations presented in Figure 6, in comparison to experimental data**.

		**Experiments**	**Simulations**			
			**HE SCMs**	**LE SCMs**	**1/3 HE SCMs + 2/3 LE SCMs**	**1/3 HE SCMs + 2/3 LE SCMs with enhanced heterocellular coupling**
Quadratic fit a_2_· p^2^_SCM_+a_1_· p_SCM_+a_0_	a_2_	7.8 (0.2-15.5)	39.3 (37.5-41.0)	34.8 (33.5-36.1)	36.1 (34.6-37.5)	17.3 (16.4-18.2)
	a_1_	−37.6 (−45.1 to −30.1)	−69.8 (−71.7 to −68.0)	−64.9 (−66.3 to −63.5)	−66.8 (−68.3 to −65.3)	−46.9 (−47.8 to −46.0)
	a_0_	34.9 (33.7-36.2)	36.2 (35.8-36.6)	35.8 (35.5-36.1)	36.1 (35.8-36.4)	35.5 (35.3-35.7)
Linear fit a_1_· p_SCM_+a_0_	a_1_	−30.2 (−32.3 to −28.1)	−30.6 (−32.1 to −29.0)	−30.1 (−31.4 to −28.7)	−30.7 (−32.2 to −29.3)	−29.6 (−30.3 to −29.0)
	a_0_	34.4 (33.2-35.6)	30.3 (29.4-31.3)	30.6 (29.8-31.4)	30.7 (29.9-31.5)	32.9 (32.5-33.3)

*95% CIs are given in parentheses*.

**Table 4:** **Intercellular gap junctional coupling levels in the computer model with enhanced heterocellular coupling**.

**Relative coupling**	**PCM**	**LE-SCM**	**HE-SCM**
PCM	1	0.1538	0.0821
LE-SCM	0.1538	0.0385	0.0385
HE-SCM	0.0821	0.0385	0.0205

## Discussion

In this study, we have relied on an *in vitro* model to investigate whether SCMs have the ability to functionally contribute to electrical signal propagation in a way that may permit integration for future cell therapeutic applications in the diseased heart. We chose murine cell models for the major reasons of the availability of murine cardiac primary cells and thus the avoidance of species-specific cell differences. With regard to the electrophysiological profile, both cell types used in this study—SCMs derived from embryonic stem cells and PCMs derived from fetal ventricles—are well-characterized cardiac cell models sharing similar features of an immature cardiac phenotype, thus justifying a direct comparison of their functional properties.

### Intercellular coupling, cellular excitability, and conduction in cultured strands

Affirmative to their cardiogenic phenotype, SCMs formed strands of excitable cells on a patterned surface and electrical stimulation resulted in signal propagation along the cell strand, confirming the establishment of intercellular electrical connections between individual SCMs. One important advantage of our patterned strands grown on microelectrode arrays is that conduction is channeled along a predefined pathway, which permits accurate quantification of the steady state conduction velocity and heterogeneity of conduction in SCM preparations, both in comparison with pure PCMs and mixed cultures of both cell types. In unstructured two-dimensional monolayers, the accuracy of this analysis would be notably precluded during heterogeneous or irregular propagation by the appearance of distorted activation fronts and possible conduction blocks, which render the estimation of CV unreliable.

As shown previously (Gaudesius et al., 2003; Miragoli et al., 2006), electrical coupling between cardiomyocytes and (myo)fibroblasts leads to membrane depolarization of cardiomyocytes by several mV and consequently, to a less negative resting membrane potential, which affects Na^+^ channel recovery from inactivation. Since reduced Na^+^ channel availability strongly influences the excitability of the myocytes, enhanced presence of (myo)fibroblasts in cardiac cell strands would significantly limit signal propagation and reduce CV. Therefore, co-culture of SCMs with fibroblasts or myofibroblasts would be expected to reduce CV or cause conduction block in such mixed cell strands. In this study, we limited (myo)fibroblast content of PCM preparations not only by pre-plating, but also inhibited further proliferation of putatively remaining (myo)fibroblasts by application of bromodeoxyuridine (see “Methods”). In comparison, SCMs were selected for α-MHC expression using puromycin resistance and therefore, the resulting cell pool was free of any non-cardiac cells.

Interestingly, pure SCM strands presented significantly lower CVs compared to pure PCMs, corroborating the results of other research groups (Satin et al., 2004; Zeevi-Levin et al., 2005; Mureli et al., 2013). In mixed cultures, we found that increasing the amount of PCMs resulted in an increase in CV. However, in all mixed populations containing SCMs, CV was always significantly lower than in pure PCM preparations, indicative of a reduced capacity of SCMs for intercellular signal propagation, as supported by our simulations. Reduction of CV in mixed cardiac cultures was also shown in a study by Askar et al. where direct coupling of rat neonatal cardiomyocytes with mesenchymal stem cells (MSCs) resulted in a decrease of CV from 21.6 to 12.6 cm/s (Askar et al., 2013). However, in their co-culture model the reduced CV might result from the depolarizing influence of MSCs on CMs, similar to the previous report on the direct modulatory influence of MSCs on CV of atrial HL-1 cells (Mureli et al., 2013). Still, in the current study where SCMs exhibiting a resting membrane potential more negative than −75 mV have been mixed with PCMs, we propose that reduced CV is the result of reduced coupling between both cell types. These results are in accordance with our recent findings that pairs of SCMs form significantly fewer gap junctions between each other as compared to PCMs, primarily due to the strongly reduced expression of Cx43 in the former cell type at the mRNA and protein levels (Marcu et al., 2015). The functional consequence of limited gap junction formation was a strong reduction in the temporal kinetics of dye coupling in cell pairs. Extrapolating these findings to multicellular preparations, reduced Cx43 expression and thus reduced number of gap junctions linking SCMs may explain the strong reduction in CV. Consequently, co-culture of SCMs with PCMs showed enhanced CVs due to enhanced gap junction formation between PCMs and thus better overall coupling. The heterogeneous distribution of electrical connections in mixed cultures is further expressed by the coefficient of variation of conduction times, which was highest in co-cultures that consisted of 1/3 PCMs and 2/3 SCMs. Under these conditions, electrical impulse propagation along the cell strand was more inhomogeneous than in pure cultures, indicating that SCMs represented local conduction barriers within well-coupled PCMs. At neighboring SCMs and PCMs, this resulted in a meandering propagation wave as opposed to a planar and uniform wave front, indicating a strong current source-sink mismatch in mixed cell strands.

The notion that differences in gap junction formation may be causal for the heterogeneous conduction pattern of mixed SCMs and PCMs is further supported by a recent study, which demonstrated the impact of heterogeneous Cx43 expression on CV in cell strands composed of Cx43 knockout and wild type ventricular PCMs (Prudat and Kucera, 2014). These data showed that inhomogeneous expression of connexins, including other subunits such as Cx45, within a strand of PCMs resulted in significant conduction slowing, highly irregular propagation wave fronts, increased CVarCT and conduction blocks, all contributing to an increased susceptibility for arrhythmogenesis in the cardiac tissue strands. In the present study, we did not observe a conduction block. This suggests that the difference of connexin expression between PCMs and SCMs is not as striking as between wild type and Cx43 knockout ventricular PCMs (which also express very low levels of Cx45). While it would be very interesting to visualize the combination of putatively different connexins that may form heterotypic gap junctions, reliable subtype-specific connexin antibodies are unfortunately not available for immunostainings of the most relevant cardiac connexins. As mentioned above, it is known that ventricular PCMs express mainly Cx43 and only little Cx45. We have previously screened SCMs for the expression of cardiac connexins (Cx43, Cx45, Cx40, and Cx30.2) at mRNA level and found that SCMs express mainly Cx43, but, as all other tested connexins, at significantly reduced levels compared to PCMs (Marcu et al., 2015). These observations suggest that coupling between PCMs and SCMs in our study was largely and predominantly mediated by homomeric Cx43 gap junctional channels, with channels formed by other connexins providing only a minor contribution. However, mRNA expression does not reflect protein expression levels nor plasma membrane directed expression. Therefore, functional comparison of coupling still represents the best tool for investigation of gap junctional coupling. In future, it will be interesting to analyze the exact composition of gap junctions formed between SCMs alone or mixed SCM and PCM cell pairs. However, this will require a separate detailed study involving analysis of gap junction channel conductances.

Even though conduction block was not observed in our experiments, structural discontinuities and non-uniform signal propagation bear high arrhythmogenic potential by causing conduction delays favoring reentry phenomena (Chang et al., 2006; Askar et al., 2013). Such mechanisms may be causal for the tachyarrhythmia and reentry circuits that have been described in a recent study by Chong (Chong et al., 2014), who characterized the behavior of cell grafts of human ESC-CMs in non-human primate hearts. Interestingly, in a previous study by the same team (Shiba et al., 2012), the development of pacing-induced tachycardia was largely reduced by hESC-CM grafts in cryo-injured guinea-pig hearts, indicating beneficial impact of cell grafts on cardiac function after myocardial infarction. Yet, uncoupled graft regions with isolated Ca^2+^ transients and periodicity unrelated to the ECG remained detectable, highlighting the problem of host-graft coupling.

It should be noted however that CV is not only dependent on intercellular resistance but is also critically determined by cellular excitability. Therefore, we further investigated the excitability of SCMs by measuring the upstroke velocity of APs in whole cell current clamp experiments. These results revealed two sub-populations of SCMs (starting from a similar diastolic potential) showing high and low excitability with a mean difference of about 120 V/s. In single cells, AP upstroke velocity can be directly translated into the fast Na^+^ current, which is proportional to its maximum conductance, gNa_max_. The upstroke velocity strongly differed in HE and LE-SCMs, and in HE-SCMs it was closer to that of PCMs. Since SCMs with different excitabilities had similar resting membrane potentials, the difference in the AP upstroke velocity might be explained by a variability of Na^+^ channel expression in SCMs. In single PCMs, the upstroke velocity amounts to approximately 400 V/s, whereas propagated APs in multicellular preparations represent typically slower dV/dt_max_ (160–260 V/s in 4 day old culture preparations) due to the additional load component of connected downstream myocytes (Thomas et al., 2000; Kléber and Rudy, 2004). The different excitabilities of single myocytes and multicellular strands were taken into account in the computer simulations. Interestingly, Na^+^ channel function may be modulated by Cx43 in a manner that reduced Cx43 results in a reduction of Na^+^ channel expression (Boulaksil et al., 2010; Delmar, 2012; Delmar and Liang, 2012; Jansen et al., 2012), which may thus have an impact on AP propagation and arrhythmic tendencies of cardiac tissue. Therefore, the dynamic interaction between Cx43 expression, gap junction formation and Na^+^ channel function may contribute to the differential signal propagation in SCMs and PCMs and constitutes a very interesting new focus for future investigations of SCMs.

### Computer simulations of conduction

In order to investigate the impact of both intercellular coupling and myocyte excitability on conduction properties in cell strands, a detailed model of cellular architecture representing the experimental setup was used and the CVs (as measured in pure strands) as well as the different excitabilities of HE-, LE-SCMs, and PCMs were taken into account. As discussed above, we observed that CV decreased linearly with increasing proportion of SCMs. In contrast, the relationship between the proportion of SCMs and CV was non-linear in the computer simulations. While the experimental data showed linear reduction of CV with increasing proportion of SCMs, the model predicted an initial steep decline in CV until 60% of SCM content, where a minimal level of CV was then maintained with further increase in SCM content. This discrepancy indicates an additional factor in the experiment that was initially not directly represented in the computer model. The simulations revealed that the non-linearity was not influenced by differences in cell excitability, as might have been anticipated from the differences in SCM g_Na, max_. However, further simulations incorporating an enhancement of heterocellular coupling shifted the initially simulated curve toward the linear relationship representing the experimental data.

### Regulation of heterocellular coupling

One possible explanation for the experimentally observed linear CV vs. SCM content relationship is that heterocellular coupling might be upregulated in co-cultures. This hypothesis is in line with another study (Mureli et al., 2013; Smit and Coronel, 2014) in which it was demonstrated that paracrine factors secreted by PCMs upregulate Cx43 expression in SCMs. Such upregulation may thus have contributed to the accelerated conduction in our mixed cultures, albeit not to the same level as in pure PCM preparations. Further factors may be involved in the regulation of connexin expression and gap junction formation. Direct contact of low Cx43 expressing SCMs with native, high Cx43 expressing PCMs may be sufficient to recruit existing hemichannels for dense gap junction formation or even lead to the enhanced sarcolemmal expression levels of Cx43. Therefore, as discussed in a previous study, enhanced Cx43 expression may be essential for an improvement of intercellular coupling (Roell et al., 2007; Mureli et al., 2013). If merely the contact to native myocytes was sufficient to trigger the enhanced coupling between SCMs and PCMs, the non-linearity of CVarCT would also be explained simply by the presence of PCMs touching the SCMs in our *in vitro* model. In addition, different ion current repertoires in PCMs and SCMs may also contribute to this difference, and further investigations are needed to untangle the exact role of each ion channel and its modulation in SCMs vs. PCMs. Interestingly, in our previous study involving the heterocellular coupling between SCMs and PCMs in cell pairs (Marcu et al., 2015), no improvement in dye coupling was observed suggesting that gap junction formation in SCMs was not influenced by this co-culture approach. Yet, the experimental setting of cell pairs cannot be directly compared with multicellular preparations as formed by the cell strands.

### Limitations

Since adult cardiomyocytes cannot be kept in culture without undergoing considerable structural and functional remodeling, which would result in a loss of the adult phenotype, these experiments were restricted to the use of immature cardiomyocytes. Despite the fact that SCMs represent myocytes with properties of an early, immature cardiac phenotype, the *in vitro* differentiation process of stem cells into the cardiogenic lineage is very different from the *in vivo* differentiation of fetal primary myocytes. Therefore, the immature state of SCMs does not automatically correspond to the same functional phenotype of PCMs. This means that the mechanisms that keep SCMs in an immature state might deviate from the mechanisms that define immaturity in PCMs, namely induced cardiogenesis vs. natural developmental processes. SCMs represent a heterogeneous population of cardiac-like cells, which may differ in their AP characteristics, spontaneous beating activity and ion channel repertoire, including variabilities notably in *I*_Na_, the L- and T-type Ca^2+^ currents (*I*_CaL_ and *I*_CaT_) and the K^+^ currents, which stabilize the resting membrane potential (*I*_*K*1_), and the connexin expression patterns (Ma et al., 2011; van den Heuvel et al., 2014).

In a therapeutic setting, SCM grafts consisting of heterogeneous cell populations at this state of maturity may form islets of novel cardiomyocytes in, e.g., infarcted areas of the myocardium, which typically also consist of different populations of cardiomyocytes in the border zone vs. the surrounding muscle. However, an insufficient functional coupling of SCMs to native cardiomyocytes and the intrinsic automaticity of SCMs might not improve the instable electrical activity in the infarcted areas, but rather favor arrhythmogenesis. Therefore, further maturation of SCMs especially regarding enhanced formation of gap junctions may significantly enhance the therapeutic potential of SCMs in grafts. In our study, we did not test whether coupling and conduction may be enhanced by longer co-culture periods. The question whether time may lead to SCM maturation toward an adult phenotype *in vivo* thus remains to be answered. Interestingly, structural and functional maturation of SCMs may be triggered by special culture conditions, including culture in 3D spheroids, tissue strips (Martinez and Kofidis, 2011; Lundy et al., 2013; Nunes et al., 2013; Hirt et al., 2014; Beauchamp et al., 2015), or during electrical and/or mechanical stimulation, with pioneering studies claiming functional maturation even after implantation (Chong et al., 2014). However, the different strategies have not yet solved the problem of arrhythmogenicity of SCMs.

## Conclusion

In this study, we highlight the fact that CV, a critical parameter for arrhythmogenesis, is significantly lower in cultures of SCMs and may be improved by modulating intercellular coupling. Our data emphasize the importance of heterocellular coupling for functional integration of SCMs with native cardiomyocytes and set a new research focus on the improvement of intercellular communication of SCMs for future investigations.

### Conflict of interest statement

The authors declare that the research was conducted in the absence of any commercial or financial relationships that could be construed as a potential conflict of interest.
